# Discovery of the water scavenger beetle genus *Brownephilus* Mouchamps in Turkey (Coleoptera, Hydrophilidae, Hydrophilini

**DOI:** 10.3897/zookeys.53.455

**Published:** 2010-08-27

**Authors:** Mustafa C. Darılmaz, Suat Kıyak, Andrew E. Z. Short

**Affiliations:** 1 Department of Biology, Faculty of Science and Art, Aksaray University, TR-68100 Aksaray, Turkey; 2 Department of Biology, Faculty of Science and Art, Gazi University, TR-06500 Ankara, Turkey; 3 Division of Entomology, Biodiversity Institute, and Department of Ecology & Evolutionary Biology, University of Kansas, Lawrence, KS 66045, USA

**Keywords:** Aquatic beetles, Hydrophilidae, Middle East, Turkey, new combination

## Abstract

The recently described Hydrochara major İncekara, Mart, Polat, & Karaca, 2009 from Turkey is transferred to the genus Brownephilus Mouchamps. New records and habitat information are given for the species, as well as diagnostic features for separating it from the only other described member of the genus, Brownephilus levantinus Balfour-Browne. The discovery of Brownephilus in Turkey marks the first time the lineage has been found since its original description more than seventy years ago.

## Introduction

Members of the Hydrophilina (or “giant water scavenger beetles”) are common and readily collected in all biogeographic regions. Defined by their large size and prominent sternal keels, they are easily distinguished from other groups of hydrophiloids. Recently, Short (2010) presented a complete review and phylogenetic analysis of the subtribe. In addition to the description of an enigmatic new genus from Venezuela, Short (2010) elevated Brownephilus Mouchamps, 1959 from a subgenus of Hydrobiomorpha to full generic status. The taxon Brownephilus, diagnosed by having a broadly emarginated clypeo-labral margin but lacking long hairs on the antennal club, was erected for a single species hitherto known only by two specimens from “Palestine”.

In a recent review of the Hydrochara of Turkey, İncekara et al. (2009) described an unusually large new species of the genus, Hydrochara major. The authors note that the specimens represent the largest known examples of the genus Hydrochara yet described. However, the male genitalia of the species bear striking resemblance to that of Brownephilus levantinus (Balfour-Browne, 1939), which is also a very large species of similar size to Hydrochara major. We have made additional collections of Hydrochara major, including the first known females, and confirm that it is not a member of the genus Hydrochara, but a second species of Brownephilus. The species can easily be excluded from Hydrochara by possessing a broadly emarginated clypeo-labral margin (which, at the time the species was described, would have assigned the species to the genus Hydrobiomorpha; Brownephilus was not yet elevated to its current rank). The lack of long hairs on the antennal club and distinctive aedeagus further unambiguously place the species within the genus Brownephilus.

### Brownephilus major

(İncekara, Mart, Polat, & Karaca, 2009) comb. n.

Figures 1–2, 4 5

Hydrochara major  İncekara, Mart, Polat, & Karaca, 2009: 318.

#### Type locality.

Turkey: Samsun Province, Ondokuzmayıs, Fish Lake, 41°35'10"N; 36°06'42"E, 0 m elev.

**Figure 1-4.**
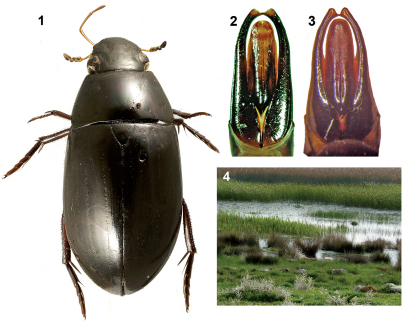
**1** Brownephilus major, dorsal habitus **2** Brownephilus major, aedeagus **3** Brownephilus levantinus, aedeagus (holotype) **4** Karakuyu Lake, Turkey, habitat of Brownephilus major.

**Figure 5:**
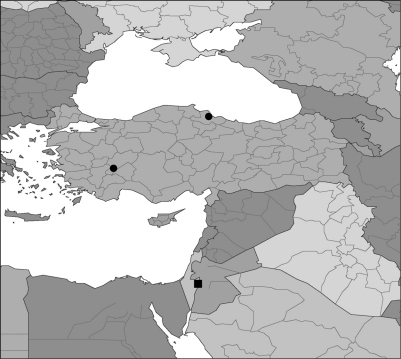
Known distribution of Brownephilus species: Brownephilus major (●); Brownephilus levantinus (■).

#### Material examined.

 TURKEY:Afyon Province: 2 ♂♂, 3 ♀♀, Dinar (Karakuyu Lake), 38°04.587"N; 30°16.505"E, 1020 m, 22.V.2009; 1 ♂, 1 ♀, same locality, 20.VI.2009 (specimens are deposited in the Gazi University Zoological Museum, Ankara, Turkey, and the Snow Entomological Collection, University of Kansas, Lawrence, USA).

#### Differential diagnosis.

 Total body length 20.0–21.5 mm. Very similar to Brownephilus levantinus, from which it may be distinguished by the shape of the aedeagus: the outer margins of the parameres are slightly sinuate medially, with the apex noticeably prolonged apically in Brownephilus levantinus (Fig. 3) while they are straight with the apex only slightly prolonged apically in Brownephilus major (Fig. 2).

#### Biology.

All collecting events for this species were from the margins of moderately to densely vegetated, standing waters (Fig. 4).

## Discussion

The rediscovery of the genus Brownephilus is significant as its taxonomic and phylogenetic placement has been enigmatic, the genus was known from only two slightly damaged specimens, and nothing was known of its biology or distribution.

The locality where we recollected the genus (Karakuyu Lake) is included in the Ramsar List of Wetlands of International importance under the UNESCO’s Convention on Wetlands of International importance especially as waterfowl habitat. The lake, with a total area of 1220 ha and a maximum depth of 3.5 m, is fed by both surface and groundwater (Nergiz and Tabur 2007).

## Supplementary Material

XML Treatment for Brownephilus major
